# Failure to Guard: Mitochondrial Protein Quality Control in Cancer

**DOI:** 10.3390/ijms22158306

**Published:** 2021-08-02

**Authors:** Joseph E. Friedlander, Ning Shen, Aozhuo Zeng, Sovannarith Korm, Hui Feng

**Affiliations:** 1 Department of Pharmacology and Experimental Therapeutics, Boston University School of Medicine, Boston, MA 02118, USA; jfriedla@bu.edu (J.E.F.); shenning@bu.edu (N.S.); az5@bu.edu (A.Z.); ksnarith07@gmail.com (S.K.); 2 Department of Medicine, Section of Hematology and Medical Oncology, Boston University School of Medicine, Boston, MA 02118, USA

**Keywords:** mitochondria, MPQC, proteostasis, chaperone, protease, cancer, tumorigenesis, oncogene, tumor suppressor, therapeutic targeting

## Abstract

Mitochondria are energetic and dynamic organelles with a crucial role in bioenergetics, metabolism, and signaling. Mitochondrial proteins, encoded by both nuclear and mitochondrial DNA, must be properly regulated to ensure proteostasis. Mitochondrial protein quality control (MPQC) serves as a critical surveillance system, employing different pathways and regulators as cellular guardians to ensure mitochondrial protein quality and quantity. In this review, we describe key pathways and players in MPQC, such as mitochondrial protein translocation-associated degradation, mitochondrial stress responses, chaperones, and proteases, and how they work together to safeguard mitochondrial health and integrity. Deregulated MPQC leads to proteotoxicity and dysfunctional mitochondria, which contributes to numerous human diseases, including cancer. We discuss how alterations in MPQC components are linked to tumorigenesis, whether they act as drivers, suppressors, or both. Finally, we summarize recent advances that seek to target these alterations for the development of anti-cancer drugs.

## 1. Introduction

**Figure 1:**
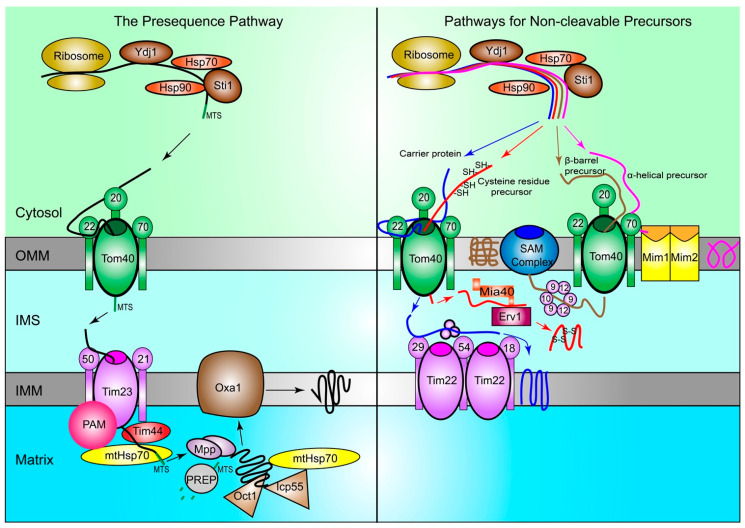
Proteins are imported into mitochondria through multiple pathways. In yeast, protein precursors are synthesized by ribosomes in the cytosol and guided to the TOM complex on the OMM by chaperone Hsp70 and Hsp90, with the help of co-chaperones Ydj1 and Sti1. In the presequence pathway (left), precursors are first recognized by Tom20 of the TOM complex in the cytosol. Then, precursors are handed to Tom22 and subsequently brought through the Tom40 channel into the IMS. They are received by Tim50 of the TIM23 complex on the IMM and passed through to the matrix, with the help of the Tim44 protein and mtHsp70. The PAM complex prevents precursors from moving backwards out of the TIM23 complex. MPP cleaves the MTS to release proteins. Icp55 and Oct1 help remove any unstable amino acids from the N-terminal cleavage site of the proteins. Then, the presequence is degraded by PREP, and Oxa1 helps integrate mature proteins into the IMM when needed. In the pathways for non-cleavable proteins (right), carrier proteins (blue), proteins with cysteine residues (red), α-helical (purple), and β-barrel (brown) are shown. Carrier proteins pass through the TOM complex via the Tom40 channel into the IMS. Then, they are carried to their final location in the IMM by the TIM22 complex and other IMS chaperones. Precursors with cysteine residues also pass through the TOM complex via the Tom40 channel. Then, they are received by Mia40, which is regulated by Erv1, and helps create the disulfide bonds between cysteine residues and form mature proteins. α-helical precursors rely on Tom70 for recognition and do not localize to the IMS, as they are integrated into the OMM by the MIM complex. β-barrel precursors pass through the Tom40 pore into the IMS, and then moved to the SAM complex on the OMM via Tim chaperones, including Tim9, Tim10, and Tim12. Then, the SAM complex incorporates the mature proteins into the OMM. TOM: translocase of the outer membrane; OMM: Outer mitochondrial membrane; Hsp70: Heat-shock protein 70; Hsp90: Heat-shock protein 90; Ydj1: Yeast dnaJ 1; Sti1: Stress inducible protein 1; IMS: Intermembrane space; TIM: translocase of the inner membrane; IMM: Inner mitochondrial membrane; PAM: Presequence translocase-associated motor; MPP: Mitochondrial processing peptidase; MTS: Mitochondrial targeting sequence; Icp55: Intermediate cleaving peptidase 55; Oct1: Octapeptidyl aminopeptidase; PREP: Presequence peptidase; Oxa1: Mitochondrial inner membrane protein OXA1; Mia40: Mitochondrial intermembrane space import and assembly protein 40; Erv1: FAD-linked sulfhydryl oxidase ERV1; MIM: mitochondrial import complex; SAM: Sorting and assembly machinery.

Long known as the powerhouse of the cell, mitochondria are the central hub of cellular metabolism, homeostasis, and stress responses, regulating cell growth, division, differentiation, and death [1,2]. In humans, mitochondria are passed down to offspring through the fertilized egg, with the initial mitochondrial DNA (mtDNA) primarily derived from the mother [3,4]. Mitochondria contain an outer (OMM) and an inner (IMM) membrane, facilitating the formation of two aqueous compartments: the intermembrane space (IMS) and the matrix (Figure 1). Nuclear DNA (nDNA) encodes approximately 99% of mitochondrial proteins with the remaining 1% from mtDNA [5]. Most oxidative phosphorylation (OXPHOS) components and the proteins required for metabolism and biogenesis are transcribed in the nucleus, subsequently translated by ribosomes in the cytosol, and finally imported into mitochondria for proper folding and assembly [6]. The remaining 13 transmembrane components of OXPHOS, which are encoded by mtDNA, are transcribed and translated in the mitochondrial matrix, and then assembled and inserted into the IMM [7].

Since proteins in mitochondria are derived from different cellular compartments, they face a unique set of challenges to maintain homeostasis. Compared to the cytosol, mitochondria possess higher reductive potentials, pH, and temperature [8]. Mitochondrial proteins are prone to errors during folding and assembly due to oxidative stress and post-translational modifications [9,10,11,12,13]. To maintain proteostasis, the cell exploits the mitochondrial protein quality control (MPQC) machineries to prevent, correct, and eliminate the mistranslated, mislocated, and/or misfolded proteins. A compromised MPQC often leads to mitochondrial dysfunction, which is commonly associated with human diseases including cancer [14]. An in-depth knowledge of MPQC is crucial to elucidate disease pathogenesis, especially tumorigenesis.

Cancer is one of the leading causes of death worldwide. Among the hallmarks of cancer, altered mitochondrial functions contribute to cancer initiation, progression, and treatment resistance [14]. Mitochondrial dysfunction can result from genetic mutations in nDNA and mtDNA, defects in gene transcription, mistakes in protein translation and import, or errors in post-translational modifications [15,16]. In the past three decades, significant efforts have been employed to investigate the role of MPQC in maintaining mitochondrial integrity and the pathologic consequences due to its disruption. Alterations of the MPQC pathways have been detected in various types of cancer, with some attributed to tumorigenesis [17]. Here, we summarize current knowledge on mitochondrial proteostasis and its quality control systems, with a focus on how the dysregulation of these processes contributes to tumor development and the potential to exploit this knowledge for therapeutic intervention.

## 2. The ORIGIN of Mitochondrial Proteins

Mitochondria in eukaryotes are commonly believed to arise from the engulfment of an aerobic prokaryote by a single-cell organism with a nucleus, under the rising levels of oxygen in the Earth’s atmosphere and the need for symbiosis between early anaerobic and aerobic organisms [18,19]. Upon being engulfed, the aerobic organism’s genes were kept separately (i.e., mtDNA inside the mitochondria) to maintain the proper localization of their encoded proteins, which are highly hydrophobic [20].

### 2.1. Mitochondrial Proteins Encoded by nDNA

In humans, nearly 1500 mitochondrial proteins are translated by cytosolic ribosomes from mRNA encoded by nDNA [21]. These ribosomes reside in the vicinity of the OMM or near contact sites between the mitochondria and the endoplasmic reticulum (ER) [22,23]. During protein transport from cytosol to mitochondria, chaperones and co-chaperones play a critical role to prevent the misfolding or damage of mitochondrial precursor proteins [24]. Co-chaperones, including Yeast dnaJ 1 (Ydj1; the homolog of human heat-shock protein 40 (HSP40)), first target unfolded precursor proteins, which are subsequently transferred to Hsp70 and then to Hsp90 [25,26]. This transfer step is also facilitated by the co-chaperone stress inducible protein 1 (Sti1), the yeast homolog of HSP70/HSP90 organizing protein, leading the precursors to the translocase of the outer membrane (TOM) complex (Figure 1) [27,28,29,30,31]. Mistakes during this process can trigger the degradation of these precursors by the cytosolic ubiquitin–proteasome system [32]. The transport of these precursors, those with and without a cleavable presequence, from cytosolic ribosomes to mitochondria is summarized below.

#### 2.1.1. The Presequence Pathway

Precursor proteins with an N-terminal mitochondrial targeting sequence (MTS) make up more than 60% of mitochondrial proteins and utilize the presequence pathway (Figure 1) [21,33]. The chaperones and co-chaperones in the cytosol bind to precursors to prevent their folding and facilitate their recognition by Tom20 of the TOM complex on the OMM [26]. Then, these precursors with the MTS presequence, which is positively charged, are handed to the central receptor, Tom22, and driven inward by the mitochondrial membrane potential and the negative charges inside the mitochondrial matrix [26,33,34].

Subsequently, precursors pass through the Tom40 pore and are moved to the translocase of the inner membrane 50 (Tim50), which is a subunit of the TIM23 complex in the IMM. The next step is to transfer precursors from Tim23 to the presequence translocase-associated motor (PAM) via the coupling activity of Tim44 [34]. PAM helps move precursors into the matrix, thus preventing them from returning to the cytosol [6]. Then, precursors are driven through the IMM, facilitated by the high membrane potential and the ATP-dependent actions of the mitochondrial Hsp70 (mtHsp70) [32]. Then, the MTS presequence is cleaved by the mitochondrial processing peptidase (MPP) as mitochondrial chaperones stabilize the premature protein [35]. Intermediate cleaving peptidase 55 (Icp55) and octapeptidyl aminopeptidase (Oct1) remove any unstable amino acids from the N-terminal cleavage site of the premature protein [34]. The cleaved N-terminal MTS presequence is subsequently degraded by the matrix peptidasome, presequence peptidase (PREP) [36], as chaperones within the matrix, such as mtHsp70, help with further folding and maturation of the protein [34]. If the destination of the precursor is not in the matrix, the translocase Oxa1 inserts it into the IMM [37].

#### 2.1.2. Pathways for Non-Cleavable Precursors

Precursors that are non-cleavable include carrier proteins as well as those with cysteine residues, α-helixes, or β-barrels (Figure 1). After they are synthesized in cytosol, carrier precursors are first recognized by Tom70 and then handed over to the general central receptor Tom22 before passing through the Tom40 pore [6]. The Tim22 complex receives these precursors through their interaction with the Tim29 subunit [38,39]. The Tim22 complex binds to carrier precursors and guides them to their destination typically via small Tim chaperones in the IMS, which inserts them into the IMM when the membrane potential is high [31,39,40].

The recognition of precursors with cysteine residues is facilitated by the mitochondrial intermembrane space import and assembly protein 40 (Mia40) after the precursors pass through the Tom40 pore. Mia40 aids in the formation of the disulfide bonds between cysteine residues during protein folding [26], which is crucial for the proper import and assembly of these precursors in the IMS [41]. The function of Mia40 is mediated by its reduction and oxidation through the sulfhydryl oxidase Erv1, which also prevents the protein from backsliding out of the mitochondria [40].

The import pathways for precursors possessing α-helixes or β-barrels differ slightly from those with cysteine residues. α-helical precursors, which make up more than 90% of integral proteins of the OMM, are first recognized by Tom70 but do not go through the Tom40 pore. The mitochondrial import (MIM) complex, which consists of Mim1 and Mim2, interacts with the Tom complex to integrate α-helical precursors into the OMM [42]. β-barrel precursors are imported through the Tom40 pore, with small Tim chaperones guiding them to the OMM-bound sorting and assembly machinery (SAM) complex. The SAM complex, specifically the Sam50 protein, integrates β-barrel precursors into the OMM [43,44].

### 2.2. Mitochondrial Proteins Encoded by mtDNA

Different from nDNA, mtDNA is circular and only ≈16.5 kb, encoding 2 mitoribosomal RNAs (mt-rRNAs), 22 mitochondrial transfer RNAs (mt-tRNAs), and 13 hydrophobic subunits essential for OXPHOS [45]. Prior to protein biosynthesis from mtDNA, the relevant regulators encoded by nDNA must be imported into mitochondria and folded into their matured states. This process is critical for the maintenance of mtDNA, as well as the biosynthesis, modification, and stability of mt-tRNAs and mitochondrial proteins [46]. Activated auxiliary proteins, such as phosphorylated mitochondrial transcription factor A (TFAM) and mitochondrial transcription factor B2, recruit DNA-dependent RNA polymerases to initiate mtDNA transcription [47]. Then, the elongation of mitochondrial mRNA (mt-mRNA) is achieved and maintained by transcription elongation factors [48]. Upon transcriptional termination, mt-mRNA are released and processed through intron excision and polyadenylation in the matrix [49].

Protein translation in mitochondria follows the same major steps as that in cytosol: initiation, elongation, termination, and ribosome recycling [50]. In the matrix, mitochondrial ribosomes (i.e., mitoribosomes) translate mature mt-mRNA into functional proteins. Similar to the cytosolic ribosome, the mitoribosome possesses a large (mt-LSU) and a small (mt-SSU) subunit, each consisting of an aminoacyl-(A-), peptidyl-(P-), and exit-(E-) site [51,52]. However, mitoribosomes have a lower sedimentation coefficient, molecular weight, and number of associating rRNAs [53]. Due to the origin of mitochondria, the protein components of mitoribosomes are similar to those of bacteria [50].

Translation is initiated when a mitochondrial initiation factor, mtIF2 or mtIF3, binds to both the start codon of mature mt-mRNAs and the P-site in mtLSU [54]. The binding triggers the recruitment of charged mitochondrial methionyl transfer RNA (mt-tRNA^Met^) to the P-site, which symbolizes the start of translational elongation [55]. Other mitochondrial elongation factors—elongation factor Tu, Ts, and G—are subsequently recruited to the mitoribosomes to facilitate elongation [47]. All 13 mtDNA-encoded peptides have long stretches of hydrophobic residues and are destined to the IMM. To avoid the aggregation and precipitation of hydrophobic residues in the matrix, nascent peptides are inserted into the IMM as they are synthesized [56]. Similar to the presequence pathway, this co-translational insertion is facilitated by Oxa1 insertase and mitochondrial ribosomal protein L45 on the IMM [7,37]. During translational termination, the mitochondrial release factor recognizes and binds to the stop codon of mRNA and the mitoribosomal A-site, which triggers the hydrolysis of the peptidyl-tRNA bond and subsequent exit of mature proteins from mitoribosomes. Lastly, mitoribosomal recycling factors catalyze the release of the mRNAs, tRNAs, mt-LSUs, and mt-SSUs [57,58].

Activator proteins, such as translational activator Pet309 in yeast, play a critical role in regulating mitochondrial protein synthesis [7]. These activator proteins interact with specific mt-mRNAs in the 5′ untranslated regions (5′-UTR) and both subunits of mitoribosomes. On the other hand, most mammalian mt-mRNA lack 5′-UTR. Therefore, activator proteins (e.g., translational activator of cytochrome C oxidase I) either stabilize mtDNA-encoded peptides or interact with translation termination factors to prevent the premature release of the peptides [59].

## 3. MPQC: Guardians of the Mitochondrial Proteome

**Figure 2:**
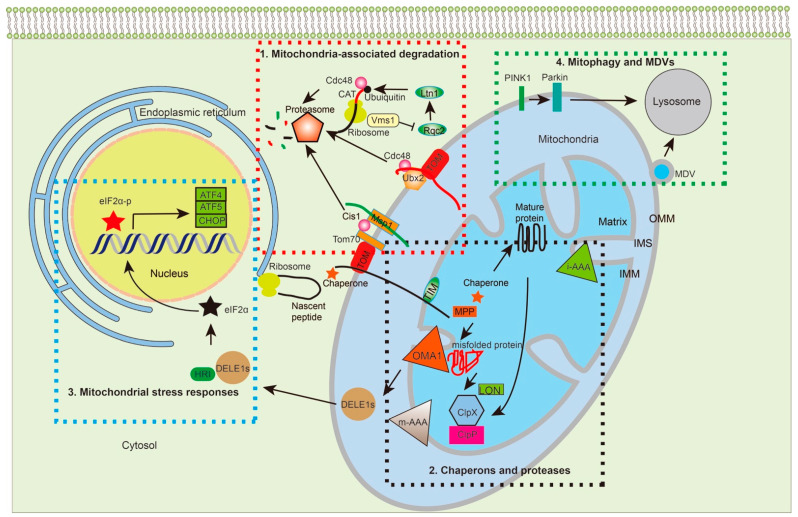
Multiple MPQC pathways surveil protein synthesis, import, folding, assembly, and degradation. The MPQC surveillance system can be divided into four pathways based on studies from yeast and humans: 1. Translocation-associated protein degradation. Under standard culture conditions of yeast, Ubx2 recruits Cdc48 to the TOM complex to target prematurely folded, misfolded, or clogged proteins for proteasome-mediated degradation. Under stress conditions, the cytosolic protein Cis1 links Msp1 to Tom70, enabling Msp1 to extract the target proteins from the TOM complex for degradation. When proteins are stalled at ribosomes, Rqc2 adds CAT-tails to the stalled proteins and facilitates their ubiquitylation by Ltn1. Vms1 removes stalled proteins for degradation through preventing Rqc2 to add CAT-tails. 2. Chaperones and proteases: Chaperones and proteases are important for protein transport, folding, and degradation. Chaperones, such as Hsp70, bind to the newly synthesized proteins and prevent their folding before transporting into mitochondria. Upon transportation into the matrix, MTS is cleaved off by MPP, with mtHsp70 working with Hsp60 to further fold the protein into its functional state. m-AAA and i-AAA are located on the OMM to remove damaged proteins under stress. In the matrix, the proteases, Lon and Plpxp, regulate protein turnover and remove damaged proteins that are accumulated under stress. 3. Mitochondrial stress responses: In humans, the i-AAA protein OMA1 cleaves DELE1 into multiple DELE1s, which interact with HRI to phosphorylate eIF2α. The eIF2α-p further regulates gene expression, such as ATF4, ATF5, and CHOP, the upregulation of which further enables the coping of the stress response. 4. Mitophagy and MDVs: When mitochondrial dysfunction persists and proteostasis cannot be restored, the accumulation of dysfunctional polypeptides together with misfolded and unfolded proteins imposes a severe burden onto mitochondria. Under such conditions, PINK1 phosphorylates Parkin, which ubiquitinates the OMM for mitophagy. MDVs serve as another strategy to remove aberrant mitochondrial proteins without losing the whole organelle. TOM: translocase of the outer membrane; TIM: translocase of the inner membrane; UPS: ubiquitin-proteasome degradation system; MDVs: mitochondria-derived vesicles; PINK1: phosphatase and tensin homolog (PTEN)-induced kinase; Parkin: an E3 ubiquitin-protein ligase encoded by *PARK2*; i-AAA: inner membrane-embedded AAA protease; m-AAA: matrix-embedded AAA protease; MPP: matrix processing peptidase; OMA1: overlapping activity with m-AAA protease; Lon: Lon protease; Clpx: ATP-dependent Clp protease proteolytic subunit X; Clpp: ATP-dependent Clp protease proteolytic subunit P; Ubx2: UBX domain-containing protein 2; Rqc2: ribosome quality control complex subunit 2; Ltn1: E3 Ubiquitin Ligase Listerin; Cdc48: Cell division control protein 48; Cis1: citrinin sensitive knockout protein 1; ATF4: Activating Transcription Factor 4; ATF5: Activating Transcription Factor 5; CHOP: C/EBP homologous protein.

While living organisms strive to accurately convey genomic information into proteins, errors in protein synthesis and import are inevitable. MPQC, the mitochondrial protein surveillance system, seeks to mitigate these mistakes through multiple mechanisms (Figure 2). In the cytosol, cells utilize the conserved ubiquitin proteasome machineries to target misfolded proteins or those stuck to the OMM for degradation [60]. Inside the mitochondria, chaperones and proteases team up to recognize, refold, and degrade mitochondrial proteins that are disassembled or misfolded [61]. Meanwhile, mitochondrial proteotoxic stress is communicated back to the nucleus, leading to increased transcription of chaperones and proteases. Finally, when beyond repair, the cell sends part of the mitochondrial membrane as secreted vehicles or whole mitochondria to lysosomes for elimination (i.e., mitophagy) [62,63,64]. All of these MPQC surveillance mechanisms are critical for mitochondrial proteostasis and the overall health of the cell.

### 3.1. Mitochondria-Associated Degradation Pathways

Mitochondrial proteins are primarily synthesized in the cytosol and then transported into their respective compartments where they execute their functions. The normal importing process is illustrated in sections above and in Figure 1. Defects in this process result in the mislocalization of mitochondrial proteins in the cytoplasm or OMM. Despite the efforts of cytosolic chaperones and co-chaperones in preventing protein aggregation, some precursors still become prematurely folded or misfolded, leading to both failure to pass through the TOM complex and a clogged channel on the OMM [31,65]. In yeast, defects in mitochondrial protein import lead to enhanced proteasomal activity and degradation of these precursor proteins in the cytosol [31,60]. Specifically, they employ three MPQC pathways: (1) mitochondrial protein translocation-associated degradation (mitoTAD), (2) mitochondrial compromised protein import response (mitoCPR), and (3) mitochondria-localized ribosome-associated quality control (mitoRQC).

#### 3.1.1. MitoTAD

MitoTAD serves as an MPQC pathway to clear trapped precursors from the TOM channel under non-stressful, standard growth conditions, using UBX domain-containing protein 2 (Ubx2) as the key regulator. Ubx2 normally functions in the Doa10 and Hrd1 E3 ubiquitin complexes, serving as an adaptor for the Ufd1–Cdc48–Npl4 complex in endoplasmic reticulum-associated degradation [66,67]. On the OMM, Ubx2 binds to Tom40 without interacting with Doa10 and Hrd1, utilizing its UBX domain to recruit the Ufd1–Cdc48–Npl4 complex to the TOM complex [65]. The co-factors, Ufd1 and Npl4, facilitate the association of Cdc48 with the TOM complex and work together with Cdc48 to remove clogged precursors for proteasome-mediated degradation under standard growth conditions [65].

#### 3.1.2. MitoCPR

Although the mitoTAD pathway can efficiently monitor the TOM complex to prevent clogging of the import channel, this process does not work well under stress conditions. Pathological conditions, such as decreased membrane potential induced by OXPHOS inhibitors or the overexpression of hydrophobic-fragment-containing proteins that are prone to clog the TOM channel, activate mitochondrial precursor overaccumulation stress (mPOS) [68]. Under the mPOS, the target of rapamycin (TOR) signaling is inhibited, leading to reduced import of mitochondrial proteins. Anti-degenerative genes, such as Zinc finger protein Gis2 and Nucleolar GTP-binding protein 2 (Nog2), are upregulated in response to mPOS to promote cell survival. Mistargeting of mitochondrial proteins can also induce an unfolded protein response activated by mistargeting of proteins (UPRam), leading to the upregulation of certain proteins [69]. For example, the overexpression of mistargeted proteins Pet191 and Mix17 significantly increased proteasomal activity while reducing protein synthesis. Hence, both mPOS and UPRam inhibit protein translation and seek to reduce the proteotoxic stress induced by proteins that fail to import into mitochondria.

When traveling from the cytosol to the OMM, mitochondrial protein precursors are recognized by the channel-forming protein Tom40 and its three small Tom partners (Figure 1) [70,71,72,73]. These three Tom receptors possess partially overlapping specificities and can substitute for each other in recognizing substrates. When one receptor is defective, the other two receptors can facilitate the import of precursor proteins, albeit with reduced efficiency [74]. In yeast, the mitochondrial comprised protein import response (mitoCPR) is activated by proteotoxic stress resulting from unimported proteins. During mitoCPR, the transcription factor heat shock factor 1 (Hsf1) can upregulate the expression of chaperones and proteases to enhance MPQC and reduce proteotoxic stress [75]. In addition, pleiotropic drug resistance protein (Pdr3) is responsible for the transcriptional reprogramming during mitoCPR [76]. For instance, the transcription factor Pdr3 activates expression of the cytosolic protein citrinin sensitive knockout protein 1 (Cis1) [76]. Then, the cytosolic Cis1 recruits AAA ATPase Mitochondrial Sorting of Protein 1 (Msp1) to Tom70 and targets stalled proteins from the translocase for proteasome-mediated degradation [76]. Msp1 also extracts mistargeted ER and peroxisomal tail-anchored proteins from the OMM [77,78,79]. However, whether mitoCPR exists in higher eukaryotes remains unclear. 

#### 3.1.3. MitoRQC

If faulty proteins become stuck at ribosomes during translation, the RQC removes the stalled protein and promotes its degradation [80]. In yeast, the ribosome quality control complex subunit 2 (Rqc2) and a ubiquitin E3 ligase Ltn1 are the key regulators in preventing the import of faulty polypeptides [81]. Specifically, Rqc2 recruits the E3 ubiquitin ligase listerin (Ltn1) to ubiquitinate the stalled polypeptides on the ribosome. Subsequently, the ubiquitinated peptides are removed from the 60S ribosome by Cdc48 for proteasome-mediated degradation. If lysine residues of the faulty polypeptides are buried in the ribosome and are not available for ubiquitylation, Rqc2 adds alanine and threonine (CAT) tails to the ribosome-stalled polypeptides, enabling the exposure of the buried lysine residues. However, the CAT-tailed nascent chains can form aggregates in the cytosol or mitochondria after being imported. As an alternative mechanism, the peptidyl-tRNA hydrolyase Vms1 can also dock on the ribosome to release the stalled peptides for proteasome-mediated degradation. Hence, Vsm1′s binding to the ribosome can prevent Rqc2 from recruiting Ltn1 and minimize Rqc2′s ability to add the CAT tails to the nascent polypeptides [82,83].

### 3.2. Chaperones and Proteases: Guarding Proteins Inside Mitochondria

MPQC inside the matrix is relatively independent from its cytosolic counterpart. It is well equipped with numerous chaperones and proteases to facilitate the proper folding, functional assembly, and correct localization of proteins. When proteins are misfolded or unfolded, proteases inside the mitochondrial matrix cleave them to clear out these faulty proteins.

#### 3.2.1. Chaperones

Chaperone proteins represent one major class of enzymes in MPQC [84]. The chaperone families include Hsp40, Hsp60, Hsp70, Hsp90, Hsp100, and sHsp (small Hsp), which are all ATPases and named based on their estimated molecular masses [85]. In this section, we mainly focus on Hsp70, Hsp60, and Hsp90, which are critical players in MPQC.

Prior to and during protein import, precursors need to remain in an unfolded state in order to pass through the small translocase channels, which is facilitated by the highly conserved molecular chaperone Hsp70. Hsp70 binds to the cytosolic precursors via its substrate binding domain until these precursor proteins are captured by the specific protein machineries, which deliver them to the translocation channels on the OMM [86]. After reaching the desired compartment, imported proteins need to be refolded to achieve their functional conformation. mtHsp70 works closely with Hsp60 and other chaperones, such as J-family co-chaperones, to fold the imported proteins and thus prevent their aggregation [87,88,89]. The coordinated efforts of chaperones and co-chaperones in protein import, folding, and assembly minimize the accumulation of non-functional proteins and ensure the formation of proper protein complexes.

Hsp60 and its co-chaperone Hsp10 form a symmetric double-ring structure and are typically located inside the mitochondria. This protein folding apparatus can interact with and accommodate unfolded polypeptide chains up to 60 kDa [90,91]. While Hsp60 binds to the unfolded proteins [92], Hsp10 acts on the outer border of the heptameric ring to open and close the ring, which regulates both the interactions of the Hsp60 monomers and its ATP hydrolysis [93].

Acting downstream of Hsp70, Hsp90 is localized inside the mitochondrial matrix to assist protein folding [94]. In bacteria, TPR domain-containing family Hop uses multiple TPR domains to bind to Hsp70 and Hsp90 simultaneously. Both bacterial Hop and its yeast homolog (i.e., Sti) can promote Hsp90-stimulated refolding in rabbit reticulocyte lysate [95]. Interestingly, a recent report indicates that the Hsp70–Hsp90 cascade does not fold proteins directly; instead, it prepares proteins for spontaneous, productive folding. At physiological concentrations, Hsp70 stalls protein folding by binding to their hydrophobic, core-forming segments. However, Hsp90 can break this deadlock and restart the folding process [96]. Regardless, neither Hsp70 nor Hsp90 alters the folding rate despite ensuring high folding yields.

#### 3.2.2. Proteases

Another group of enzymes in MPQC is the proteases, which are responsible for the protein turnover and processing. The protein density on the IMM is particularly high, enabling it to appropriately respond to nutrient and oxygen supply. Two hexameric AAA proteases, i-AAA and m-AAA, play a central role in surveilling proteins on the IMM. The name of these two proteases is derived from the fact that the catalytic domain of human m-AAA protease homologs - AFG3-like protein 2 and Paraplegin - faces the matrix while the catalytic domain of the i-AAA protease homolog - ATP-dependent zinc metalloprotease YME1L1, overlapping with the m-AAA protease 1 (OMA1) and mitochondrial inner membrane protease ATP23 homolog - intervenes in the IMS. Both proteases contain an AAA domain, which delivers substrates to their proteolytic center [97,98]. These membrane AAA proteases have several important roles, the most important of which is to control the quality of mitochondrial membrane proteins, especially for the respiratory chain proteins on the IMM [99,100]. In addition, under stressful conditions, the i-AAA protease YME1L removes the TIM17A protein from the OMM, thus reducing protein import into the mitochondria [101]. YME1L and OMA1 can also cleave Optic atrophy protein 1 (OPA1), generating the short form of OPA1 to regulate mitochondria fission [102]. However, when ATP is depleted under mitochondrial stress and the IMM is depolarized, the ATP-independent protease OMA1 can degrade YME1L, impairing the proteolytic processing of OPA1 [103,104].

In humans, there are two AAA proteases, CLPXP and LON protease (LON, Pim1 in yeast), inside the mitochondrial matrix, which degrade misfolded proteins and prevent protein aggregation [105,106]. CLPXP consists of the conserved ATP-dependent Clp protease proteolytic subunit ClpP protease and the chaperone ATP-dependent Clp protease ATP-binding subunit ClpX that functions as an unfoldase and delivers substrates in an ATP-dependent manner to ClpP [107]. In bacteria, chaperones such as ClpA and ClpC function similarly to ClpX to regulate the substrate interaction [108]. The yeast mitochondria lack ClpP and mainly depend on Pim1 for processing misfolded proteins. When misfolded and unfolded proteins in mitochondria trigger the UPR, the expression of ClpP and other chaperones increase accordingly [109].

In bacteria, Lon protease is a well-known proteolytic enzyme with three functional domains: the catalytic protease domain, the ATP-binding domain, and the N-terminal substrate binding domain [110,111]. Homologs of the Lon protease are also found in eukaryotes as one of the major enzymes exerting proteolytic activities in mitochondria [112]. For instance, Lon removes the metabolic enzyme aconitase under stressful conditions, which is prone to oxidative stress damage, thus minimizing its accumulation as aberrant protein aggregation in the matrix [113].

### 3.3. Mitochondrial Stress Responses

The main cause of mitochondrial dysfunction is impaired protein transport, which leads to the accumulation of precursors in the cytosol and on the OMM. The proteotoxic stress will trigger the previously mentioned mitochondrial protein import-associated degradation pathways, such as mitoTAD and mitoCPR. Meanwhile, the proteotoxic stress induced by these non-imported precursor proteins can activate the mitochondrial unfolded protein response (UPR^mt^), which subsequently upregulates the expression of genes encoding mitochondrial chaperones and proteases [31]. In *C. elegan*s, the UPR^mt^ is regulated by transcription factor stress activated transcription factor Atfs-1, the activating transcription factor 5 (ATF5) in mammalian cells, which contains both an MTS and a nuclear localization sequence (NLS) [114]. Under normal conditions, Atfs-1 is imported into the mitochondrial matrix and degraded by the protease Lon. Under conditions of mitochondria dysfunction, Atfs-1 import is reduced with protein accumulation in the cytosol. Atfs-1 utilizes its NLS to travel to the nucleus, activating downstream transcription [115]. Specifically, Atfs-1 induces the expression of genes encoding UPR^mt^-relevant proteins, such as the mitochondrial chaperone Hsp60 and the protease ClpP, as well as over 500 genes that impact diverse cellular activities including mitochondrial protein homeostasis, protein import, innate immunity, and mitochondrial metabolism [115,116,117].

In humans, ATF5, the ortholog of Atfs-1, regulates gene expression in the UPR^mt^. ATF5, with two other transcription factors ATF4 and CHOP, are involved in the integrated stress response (ISR), which is a general stress response pathway that modulates protein biosynthesis [118]. Four ISR kinases have been discovered to phosphorylate eukaryotic initiation factor 2α (eIF2α), including general control non-depressible 2 (GCN2), heme-regulated inhibitor (HRI), PKR-like endoplasmic reticulum kinase (PERK), and protein kinase R (PKR) [119]. The phosphorylated eIF2α further results in the suppression of global protein synthesis, while increasing the translation of mRNA with small upstream open reading frames in the 5′ untranslated region, including ATF4, ATF5, and CHOP [120,121,122].

Despite the above findings, how mitochondrial stress is linked to this general stress response pathway is not entirely clear. Recent studies show that mitochondrial dysfunction activates the inner membrane protease OMA1, which then cleaves DAP3-binding cell death enhancer 1 (DELE1), which is a protein localized in the IMS. This cleaved fragment (DELE1s) is exported into the cytosol, where it interacts with HRI and induces the phosphorylation of eIF2α and subsequent changes described above [123,124,125].

### 3.4. Mitophagy and Mitochondria-Derived Vesicles (MDVs)

If the mitochondrial dysfunction persists and proteostasis cannot be restored by the strategies mentioned above, these dysfunctional polypeptides and proteins impose a heavy burden to mitochondria. Under such circumstances, cells initiate mitophagy to eliminate the damaged mitochondria [126]. In mammalian cells, the mitophagy pathway involves two proteins: PTEN-induced kinase 1 (PINK1) and the E3 ubiquitin ligase Parkin. In healthy mitochondria, PINK1 is imported via the TOM and TIM23 complexes into the IMM where the proteases, presenilins-associated rhomboid-like protein and MPP, remove the MTS of PINK1 [127,128]. Subsequently, it is translocated back into the cytosol and degraded by the proteasome. When mitochondria are damaged (e.g., through reduction of the membrane potential), PINK1 remains at the OMM bound to the TOM complex and exposes its catalytic domain toward the cytosol. Then, it phosphorylates the E3 ubiquitin ligase Parkin and ubiquitin on the OMM. Parkin is activated on the mitochondrial surface and subsequently ubiquitylates different outer membrane proteins to label the damaged mitochondria for mitophagy [126,129].

If cells cannot afford the loss of mitochondria, they will adopt an alternative mechanism, MDVs, to remove aberrant mitochondrial proteins [126,130]. The formation of MDVs, with a size ranging from 70 to 150 nm, is independent from the mitochondrial fission machinery. Through MDVs, the cell transports a distinct subset of mitochondrial proteins to peroxisomes or late endosomes/lysosomes for degradation [131]. However, the regulatory events associated with the generation of the MDVs and involvement of the MDVs in pathophysiology is not well-established and therefore requires further studies.

## 4. MPQC Goes Awry in Cancer

Despite their efforts to maintain mitochondrial proteostasis, cancer cells with genetic mutations or altered expression of MPQC components will unfortunately show compromised MPQC, leading to proteotoxicity and dysfunctional mitochondria. Early in 1920, Otto Warburg had already proposed that cancer cells are defective in mitochondrial respiration [132,133,134]. On the other hand, somatic mutations or altered expression of MPQC components in many cancer cells arise either directly or indirectly from abnormal mitochondrial respiration [132,135]. Based on their effects on tumorigenesis, these altered genes are categorized as oncogenes or tumor suppressors (Table 1). Oncogenes are those that promote tumor cell proliferation, immune evasion, genomic instability, and/or cancer invasiveness [136]. Since the inactivation of oncogenes often reduces tumor progression, they represent promising therapeutic targets for cancer treatment. In contrast, tumor suppressor genes tend to be downregulated during malignant transformation and progression, and their upregulation, in theory, would kill cancer cells [137]. Finally, some genes can also have dual effects in promoting or suppressing tumor depending on the specific context of the cancer.

### 4.1. MPQC-Associated Genes as Oncogenes

Among the regulators involved in the MPQC pathways, gain-of-function mutations or the enhanced expression of certain genes, such as chaperones and enzymes involved in mitochondrial transcription and translation, can promote tumorigenesis. Several of them are summarized as examples below, with the rest listed in Table 1.

#### 4.1.1. Genes Encoding Mitochondrial Chaperones

In addition to their role in facilitating protein import into mitochondria, several chaperones and co-chaperones are also linked with tumorigenesis. For example, HSP70 is an ATP-dependent molecular chaperone that transfers precursors from cytosolic ribosomes to the OMM (Figure 1). Similar to other heat shock proteins, HSP70 levels are elevated under cellular stress to enable cells coping with the accumulation of unfolded or denatured proteins [213]. The overexpression of *HSP70* was shown to promote the survival of malignant cells [214]. HSP70 can stabilize serine threonine kinase AKT/PKB through their physical interaction, which generates a survival signal in response to the stimulation of growth factors. In addition, Hsp70 can bind to stress-activated kinases, including apoptosis signal regulating kinase 1 (ASK1), c-Jun N-terminal Kinase JSK, and P38 kinase, to inhibit their functions. At the mitochondrial level, the concerted action of HSP70 and HSP40 blocks the translocation of BAX from mitochondria to cytosol, thus inhibiting apoptosis [215]. At the post-mitochondrial level, HSP70 binds to apoptosis protease activating factor 1 to prevent the recruitment of procaspase-9 to the apoptosome [216]. Furthermore, together with HSP40, HSP70 inhibits tumor necrosis factor-α (TNF-α)-induced neuronal cell death by interacting with Fanconi anemia complementation group C, which inhibits interferon-inducible dsRNA-dependent protein kinase, a pro-apoptotic protein [217]. Furthermore, HSP70 can also negatively impact the autophagic pathways by activating mTOR, the negative regulator of macroautophagy, and inducing AKT phosphorylation in the lysosomes [218]. Finally, enhanced HSP70 expression can also induce resistance to chemotherapy, such as the tyrosine kinase inhibitor imatinib in human chronic myeloid leukemia cells [219]. HSP70′s ability in promoting tumor cell survival and drug resistance makes its neutralizing agents promising therapeutics for leukemia, breast, prostate, gastric, endometrial, and pancreatic cancers [218].

HSP90, another ATP-dependent molecular chaperone, hands precursor proteins to TOM and facilitates the folding, maturation, and activation of a wide array of target proteins (Figure 1) [220,221]. Similar to other heat shock proteins, the upregulation of HSP90 and enhancement of its ATPase activity are observed under cellular stress including heat, nutrient absence, and oxidative stress [222,223]. When overexpressed, HSP90 plays a major role in restoring proteostasis, thereby promoting cell survival, including that of cancer cells. HSP90 facilitates the correct folding of nascent peptides and increases the reactivation rates of damaged proteins [224]. In cancerous cells, HSP90 also stabilizes mutant proteins, ranging from those with point mutations to those with structural alterations such as BCR-ABL, thus enabling malignant transformation [225].

Mortalin/mtHsp70 is the only component of the TIM23 complex harboring the ATPase activity, which facilitates the transport of nascent peptides through the IMM and their proper folding (Figure 1). Mortalin is essential for mitochondrial biogenesis due to its ability to enable ATP-driven translocation of nascent peptides through binding to the Tim44 subunit (Figure 1) [226]. The expression of mortalin is significantly higher in most cancer cells, including those of breast, ovarian, and colorectal cancer [227]. Mortalin is shown to inactivate the pro-apoptotic role of p53, which makes its overexpression sufficient to promote cancer development in both in vitro and in vivo models, one type being breast cancer [228].

#### 4.1.2. Mitochondrial Regulators of Transcription and Translation

In addition to chaperones and co-chaperones, the transcriptional and translational regulators of mitochondrial proteins can also facilitate tumorigenesis. One regulator is mitochondrial DNA polymerase gamma 1 (POLG1), a catalytic subunit of POLG, which is the only DNA polymerase known to function in human mitochondria. The normal role of POLG1 is to replicate and repair mtDNA in the matrix, serving as an essential regulator for mtDNA and general mitochondrial function. Mutations and the overexpression of *POLG1* are commonly observed in melanoma, renal carcinoma, breast, pancreatic, and colorectal cancer (CRC). Some of the common mutations in POLG1 include E1143G, T251L, P587L, and double mutant T251L/P587L [156]. When these mutations were introduced to breast cancer cell lines, they decreased mitochondrial membrane potential and increased reactive oxygen species (ROS) levels and tumor cell invasion [157]. miRNA-array analysis demonstrated a depletion of mtDNA in these POLG1 mutant cells [157,158]. Furthermore, POLG1 E1143G cells significantly increased glucose consumption rates, displaying a switch to the aerobic glycolysis signature [156].

The mitochondrial small ribosomal subunit protein BS16m (MRPS16) is in charge of both mt-SSU assembly and nick introduction to supercoiled DNA in the matrix [229,230]. The overexpression of *MRPS16* is often found in glioma tissues and promotes tumorigenesis in animal models [138]. In an in vitro study, *MRPS16* inactivation was found to suppress tumor cell growth, migration, and the invasion of human glioma cells by inhibiting the PI3K/AKT signaling [138]. In addition, MRPS16 depletion also inhibited tumor growth in mice. Thus, MPRS16 represents a promising prognostic marker and potential therapeutic target for glioma [139].

The normal function of immature colon carcinoma transcript 1 (ICT1), a matrix protein, is to induce the hydrolysis of peptidyl tRNAs from stalled mitoribosomes [159]. In 1995, the abnormal expression of ICT1 was first found in CRC and identified as a regulator of cell differentiation [138]. High ICT1 expression in tumor tissues predicts worse overall survival in CRC patients. *ICT1* knockdown by shRNA suppressed proliferation and colony formation while inducing apoptosis in multiple types of cancer cells, including those of colorectal, prostate, breast, gastric, and lung cancer [68,160,161,162,163]. Western blot analysis showed that the AMPKα, BAD, SAPK/JNK, and PARP signaling were altered upon *ICT1* knockdown. Furthermore, cell cycle regulators, including CDK1 and cyclin B1, were downregulated, while apoptotic factors, such as cleaved PARP, caspase 3, and BAX, were upregulated [68,161,162,163]. Finally, ICT1 is also a direct target of miR-205, which exerts suppressive effects on tumor metastasis [164]. Hence, inhibiting ICT1 by RNAi or small molecular inhibitors could serve as a novel therapeutic approach for a range of cancers [68,160,161,162,163].

### 4.2. MPQC-Associated Genes as Tumor Suppressors

While the hyperaction of the above MPQC genes drives tumorigenesis, other MPQC components tend to be downregulated in cancers, which include regulators inside and outside the mitochondria. In this section, we will discuss the mutations of key MPQC proteins and how these alterations impact cancer development as tumor suppressors.

#### 4.2.1. MPQC Components Outside the Mitochondria

Among MPQC regulators, the tRNA isopentyl transferase 1 (TRIT1) resides in the nucleus, cytoplasm, and matrix, working to add an isopentyl group to adenosine 37 on tRNA [231,232,233]. The addition of the isopentyl group increases the affinity of tRNA for the ribosome. TRIT1 modifies serine tRNA for cytosolic proteins, as well as tryptophan, tyrosine, serine, phenylalanine, and cysteine tRNAs for mitochondrial proteins [234]. Single-nucleotide polymorphisms of *TRIT1* are associated with cancer progression, including increased lymph node metastasis in gastric cancers [235]. One variant of TRIT1, TRIT1-F202L, was found to predict poor survival among patients with lung cancer [236]. A mouse study shows that *TRIT1* mRNA is downregulated in lung cancer cells, while its overexpression decreases the rates of lung tumor development [237]. Its role as a tumor suppressor is most likely linked with its activities in the nucleus, since its yeast homolog, tRNA dimethylallyltransferase (Mod5), is associated with tRNA transcription complexes and pre-tRNAs [235]. Although how nuclear TRIT1 suppresses human cancer remains unclear, research suggests that TRIT1 binds to tRNA in a similar manner to Mod5 [238].

The Parkin protein resides in the cytosol, serving as an E3–ubiquitin ligase. Upon activation by PINK1, Parkin targets proteins on the OMM for degradation or induces mitophagy when they accumulate [239]. Although mitophagy may exhibit a dual role in tumorigenesis in general, the mitophagy mediated by the PINK1–Parkin pathway exerts tumor suppression in a variety of cancer types [240]. Mutations in *PARK2*, the gene encoding Parkin, are prevalent in a broad spectrum of cancers, such as melanoma, gliomas, neuroblastomas, breast, lung, and colon cancer [240]. One example is that a restored expression of *PARK2* decreases the rate of tumor cell proliferation in gliomas [241]. A crucial tumor suppressive effect of Parkin is likely associated with its regulation of P53 in glucose metabolism and its ability to reverse the Warburg effect [173,240]. One of Parkin’s substrates is cyclin E, which is a critical regulator for cell cycle progression, and its degradation would lead to cell cycle arrest, thus halting cancer development [241]. Another mechanism by which Parkin suppresses tumor growth is through its ability to inhibit necroptosis, which is a programmed inflammatory cell death. Necroptosis signifies inflammation, which contributes to the development of cancers such as CRC [242]. While Parkin facilitates P53′s response to the Warburg effect and antioxidant defenses [173], its ability to ubiquitinate hypoxia-inducible factor 1α, a crucial mediator for cancer metastasis, also contributes to tumor suppression [243]. Rotenone, and a combination of mitocans with demonstrated anti-cancer effects (e.g., metformin), may be effective in targeting the PINK1–Parkin pathway of mitophagy in cancer, such as acute lymphoblastic leukemia (ALL) [244]. Compounds, including derivatives of betulinic acid, have been found to trigger ROS and PINK1–Parkin-mediated mitophagy to induce apoptosis in multi-drug-resistant cancer cells [245].

#### 4.2.2. MPQC Players inside Mitochondria

Serine B-lactamase-like protein (LACTB), a mitochondrial protease, localizes in the IMS, regulating complex I of the ETC and lipid metabolism within the mitochondria [165,176]. LACTB is downregulated in cancer cells through multiple mechanisms, including micro-RNA-mediated translational inhibition, promoter methylation, and histone acetylation [165,177,246]. The overexpression of *LACTB* negatively impacts the growth of both breast cancer and hepatocellular carcinoma cells by modulating lipid metabolism to promote cancer cell differentiation [165,166]. In CRC, the increased expression of LACTB impairs MDM2-mediated p53 ubiquitination and degradation [177]. In gliomas, a downregulation of LACTB leads to an increase in cancer cell proliferation and angiogenesis, corresponding to a poor prognosis in patients, while its overexpression does the opposite by inhibiting PCNA, MMP2, MMP9, and VEGF, which are key regulators in glioma cell proliferation [175].

OMA1, as mentioned above, is a key protease that spans the IMM and contributes to various mitochondrial functions [247,248]. Its substrates include OPA1 (an important regulator for OXPHOS and apoptosis) and PINK1 (a kinase crucial for mitophagy) [248]. Excessive OPA1 cleavage by OMA1 leads to mitochondrial fragmentation and cell death [249]. Interestingly, this mechanism could be exploited to effectively kill cancer cells as OPA1 cleavage by OMA1 can induce the release of cytochrome c [250]. Silic-Benussi et al. selectively killed T-ALL cells using compounds that increased ROS levels, thus enhancing the OMA1-OPA1 activity [251]. Targeting the ROS–OMA1–OPA1 axis could serve as a therapeutic strategy and should be investigated further [251]. The cleavage of PINK1 by OMA1 activates protein degradation on the OMM or mitophagy mediated by the PINK1–Parkin pathway. A decrease in OMA1 expression correlates with the poor prognosis of patients with breast cancer due to changes in mitochondrial proteostasis and an increase in filopodia-like structures, which promote cell migration and proliferation [174].

Adenine nucleotide translocase 1 (ANT1) resides in the IMM, catalyzing the exchange of mitochondrial ATP for cytosolic ADP. A mouse study shows that the overexpression of *ANT1* induces apoptosis and inhibits breast cancer growth by suppressing nuclear factor κB (NF-κB), which is a protein critical for DNA transcription and cytokine production [252]. In addition, dysregulated ANT1 is also linked with mitochondrial diseases such as Sengers syndrome and autosomal dominant facioscapulohumeral muscular dystrophy [253]. Recently, restoring ANT1 expression was found to sensitize rhabdomyosarcoma cells to chemotherapy [254], indicating the potential to exploit ANT1 as a target for cancer treatment.

Leucyl-tRNA synthetase 2 (LARS2) resides in the matrix to catalyze the formation of a charged leucine-tRNA, which is essential for mitochondrial translation [255]. The downregulation of LARS2 via promoter hypermethylation or allelic loss correlates to the development of nasopharyngeal carcinoma [168]. In head and neck squamous cell carcinoma, the promoter region of *LARS2* is also methylated [256]. The downregulation of LARS2 enhances the proliferation of both types of cancer cells, which is a phenotype consistent with its role as a tumor suppressor, although how it impacts tumor development requires further investigation. Pitrilysin metallopeptidase 1 (PITRM1), another protease in mitochondria, degrades oligopeptides from imported mitochondrial proteins [167]. Although PITRM1 is known to regulate the tumor suppressor Kruppel-like factor 6 gene in breast cancer, kidney renal clear cell carcinoma, and lung adenocarcinoma, further research must be conducted to study its targetability and other implications in cancer [181].

The TOM complex on the OMM could also represent a potential target for cancer treatment as each TOM protein interacts with some of the apoptosis or cancer regulators. For instance, the PINK1–Parkin mitophagy pathway recognizes the TOM complex via the role of PINK1 in detecting accumulated proteins [257]. Another example is that TOM70 is targeted by the myeloid leukemia cell differentiation protein-1, which is an inhibitor of apoptosis and therefore a driver of cancer [258]. TOM5, a protein that helps stabilize the TOM complex, was shown to bind to P53, a well-established tumor suppressor, and their interaction suppresses cancer proliferation in non-small cell lung cancer cell lines [259]. In melanoma cells, TOM20 was found to sense ROS induced by iron and promote pyroptosis, which is a type of cell death [260].

### 4.3. Dual Roles of MPQC-Associated Genes in Tumorigenesis

Among chaperones, proteases, and regulators in the MPQC pathway, many exert distinct roles either as cancer drivers or suppressors. However, in certain cases, some of the MPQC players can have dual roles in tumorigenesis.

#### 4.3.1. Chaperones

HSP40 is a cytosolic chaperone, which helps target precursor proteins from the cytosol to the OMM and facilitates protein import, folding, and degradation [24,27]. Various types of HSP40 may impart different effects on cancer cells [184]; for example, two subtypes of DNAJ (the HSP40 family) are implicated in breast cancer development, as the DNAJA subtype induces apoptosis, while the DNAJB subtype inhibits apoptosis [185]. While HSP40 has been proposed as a target in cancer, further research is needed to investigate the role of each subtype in various types of cancers [186].

HSP60 is another chaperone protein in MPQC that exhibits a dual role in tumorigenesis. HSP60 resides in the matrix to facilitate the folding and stabilization of precursor proteins that are transported into the mitochondria [195,261]. During tumorigenesis, and depending on the cancer context, HSP60 accumulates outside of mitochondria to possibly assist in protein folding and maintain the cell’s survival, as in uterine exocervix, prostate, and large bowel cancer cell models [262]. Alternatively, it may exert a pro-apoptotic role by promoting the maturation of procaspase-3, as in JURKAT and HeLa cell lines [214,217,218]. HSP60 is overexpressed in a variety of human cancers such as leukemia, neuroblastoma, breast, lung, and liver cancer [185]. The mitochondrial levels of HSP60 in pancreatic ductal adenocarcinomas are positively correlated with disease aggression and cancer cell proliferation through the ability of HSP60 to enhance ERK1/2 phosphorylation and cancer cell survival [197]. Interestingly, in hepatocellular carcinoma (HCC), HSP60 acts as a tumor suppressor by promoting cell differentiation while inhibiting the migration and invasion of HCC cells [196]. In addition, HSP60 was found to interact with HSP10 chaperone and pro-caspase 3 in the matrix of JURKAT T-ALL cells, suggesting its pro-apoptotic activities as a tumor suppressor [193]. HSP60 has already been established as a therapeutic target for treating certain cancers due to its wide upregulation and tumor-driving role [195]. However, little research has been performed to exploit the possibility of overexpressing HSP60 as a means to treat other cancers in which it functions as a tumor suppressor.

Prohibitin (PHB) is a chaperone protein conserved across many species, consisting of two subunits, PHB1 and PHB2, which form a complex in the IMM to stabilize mitochondrial proteins, including OMA1 [263]. While PHB1 is reported to be overexpressed in pancreatic, ovarian, and gallbladder cancer, its downregulation has also been detected in HCC and nasopharyngeal carcinoma [201]. In gastric cancer, PHBs can serve as both oncogenes and tumor suppressors, with either upregulation or downregulation observed in patient tumor samples [199]. As a transcriptional target of MYC, both PHB1 and PHB2 are overexpressed in a variety of cancers, including breast, prostate, colon, testicular, and skin [198]. The tumor-suppressing role of PHB is implicated in its ability to induce p53-mediated transcription and repress E2F-mediated transcriptional activity [264,265]. In addition, PHB can also block cells’ entry into the S phase of the cell cycle [199]. In particular, PHB2 can promote mitophagy by stabilizing and helping maintain the PINK1–Parkin pathway [200]. The wide involvement of PHB in cancer necessitates further research to dissect the role of each PHB subunit in specific cancer contexts, in order to devise suitable approaches for therapeutic intervention.

#### 4.3.2. Proteases and Other MPQC Regulators

Among the proteases involved in MPQC, the ATP-dependent Clp protease proteolytic subunit (CLPP) is located in the matrix with its normal function to degrade damaged or misfolded proteins [266]. One of the CLPP substrates is succinate dehydrogenase subunit A, which is a part of complex II in the ETC, implicating its role in OXPHOS [203,267]. CLPP is also involved in mitochondrial transcription and translation, since one of its substrates, the mitochondrial GTPase Era 1, can inhibit the activity of the mitoribosome [207]. In terms of its oncogenic role, Cole et al. showed that CLPP is overexpressed in a majority of acute myeloid leukemia (AML), and *CLPP* inactivation selectively killed AML cells as a result of impaired ETC activity and increased ROS [203]. CLPP is also overexpressed in breast cancer, and CLPP depletion inhibits the SRC/PI3K/AKT pathway, which is crucial for cancer cell proliferation and invasion [206]. Compounds, such as imipridones, were found to hyper-activate CLPP, which in turn led to the death of subsets of AML, cervical, breast, ovarian, and colon cancer cells [204,205]. CLPP’s role as a tumor suppressor is not as well understood, with its downregulation detected in gastric adenocarcinomas [268].

As the key subunits of the ETC complex III, the ubiquitin–cytochrome c–reductase complex core proteins 1 and 2 (UQCRC1 and UQCRC2) have been reported to be either upregulated or downregulated in various cancers. UQCRC1 and UQCRC2 are homologous to the two subunits of MPP (β and α respectively) and cleave precursors of mitochondrial proteins that are imported into the matrix [269]. UQCRC1 is overexpressed in pancreatic ductal adenocarcinomas, osteosarcomas, as well as in breast and ovarian cancer, while it is downregulated in the clear cell subtype of renal cell carcinomas, CRC, gastric, and breast cancer [208,209,210]. The overexpression of *UQCRC1* increased the growth rates of pancreatic ductal adenocarcinoma cells in vitro and in vivo through activating the eATP/P2Y2-RKT/AKT pathway [208]. UQCRC1 downregulation in CRC was found to correlate with lymph node metastasis and poor patient prognoses [210].

The oncogenic role of UQCRC2 is indicated by its overexpression in CRC and human lung adenocarcinomas, as well as its correlation with cancer invasion and metastasis. UQCRC2 also plays a role in inducing ROS, which can promote tumorigenesis, and its knockdown induces the apoptosis of CRC cells [211]. On the other hand, UQCRC2 can inhibit glioma progression as it mediates the effect of cadherin 18 on suppressing the invasion of glioma cells [212]. Despite this knowledge, drugs that specifically target UQCRC1 and UQCRC2 have not yet been developed.

The required-for meiotic nuclear division 1 homolog (RMND1) localizes in the matrix of the mitochondria and organizes the assembly of the mitochondrial ribosome [179,202]. RMND1 is a potential tumor suppressor, as it is downregulated in chronic myeloid leukemia [202]. In the estrogen receptor negative subtype of breast cancer, RMND1 expression is reduced in tumor samples associated with minor risk alleles but increased in those with major risk alleles, suggesting its possible dual role in breast cancer pathogenesis [178]. Regardless, further research is required in order to establish RMND1 as a therapeutic target for cancer treatment.

## 5. Therapeutic Exploitation of MPQC for Cancer Treatment

With the ongoing research on MPQC and its involvement in cancer, a number of drugs and compounds targeting MPQC players have been developed and evaluated for their anti-cancer effects. In this section, we categorize these drugs and synthetic compounds based on their respective targets (Table 2). Importantly, some of these drugs require the attachment of a mitochondrial targeting compound to help guide them through the mitochondrial membranes and to their target proteins. Finally, we briefly discuss some questions that have arisen from the actionable mechanisms of these drugs and compounds and the possible future directions. Drugs targeting mitophagy include LCL-461, liensinine, chloroquine, and hydroxychloroquine [270].

### 5.1. Targeting Cytosolic Chaperones for Protein Transport

2-Phenylethynesulfonamide (PES) can inhibit HSP70 by acting on its C-terminal binding domain, which disrupts its substrate binding as well as its interactions with co-chaperones [273,291]. Although PES can inhibit the transport of P53 to mitochondria and thus block P53-mediated apoptosis, its effect on HSP70 induces an aggregation of misfolded and nascent proteins, leading to tumor cell death [273,291]. PES is also shown to induce apoptosis via ROS in vivo, and clinical trials of this compound could begin soon [274,292]. Although MKT-077, another HSP70 inhibitor, has been terminated in clinical trials due to its toxicity [293], investigations into its derivatives are ongoing [294,295].

One of the HSP90 inhibitor is 17-dimethylaminoethylamino-17-demethoxygeldanamycin (17-DMAG), which is a derivative of the toxic drug, geldanamycin [272]. 17-DMAG, also known as alvespimycin, has shown promising results in preclinical and clinical studies for treating a broad spectrum of cancers, such as leukemia, melanoma, medulloblastoma, multiple myeloma, breast, ovarian, cervical, and lung cancer [271,272]. 17-DMAG competes with ATP’s binding to HSP90 and thus inhibits its chaperone function [296]. The inhibition of HSP90 by this compound leads to misfolding, ubiquitylation, and degradation of its target proteins [297,298]. Gamitrinibs are a class of mitochondrial matrix inhibitors that targets HSP90 [299]. In cancerous cells, HSP90 accumulates in mitochondria and blocks the release of cytochrome c. Hence, the binding of gamitrinibs to HSP90 unleashes cytochrome c and the apoptotic pathway. Gamitrinibs are largely studied in prostate cancer and show strong anti-cancer effects [300]. When combined with other anti-cancer drugs, such as doxorubicin, gamitrinibs exhibit strong anti-cancer activity in HeLa cells, ovary, prostate, glioblastoma, renal cell carcinoma, hepatocellular carcinoma, and lung carcinoma cells [275].

Paclitaxel, also known as Taxol as an anti-microtubule agent, exerts its anti-cancer effects primarily through the disruption of mitosis (NCI 2015). In mitochondria, paclitaxel induces apoptosis by dissipating the mitochondrial membrane potential, leading to cytochrome c release [301]. In addition, paclitaxel is shown to bind to the C-terminal ATP-binding site of HSP90, which is a region mediating macrophage activation. Interestingly, another anti-cancer drug cisplatin can also bind to the C-terminus of HSP90 [150,151,302]. A derivative of paclitaxel, nanoparticle-albumin-bound paclitaxel (*nab-*paclitaxel), exhibits reduced side effects and increased efficacy when treating cancers such as metastatic breast cancer [276]. Currently, over 3500 ongoing clinical trials are using paclitaxel to treat cancers, whether it is paclitaxel itself, derivatives such as nab-paclitaxel, or in combination with other drugs (NCT03315364 and NCT04137653).

### 5.2. Targeting Proteins within Mitochondria

Carboplatin is a second-generation chemotherapy modified from cisplatin to mitigate its side effects [277]. Both compounds are commonly employed in the clinic for treating patients with various types of cancers. These compounds possess the ability to target and disrupt nDNA via their platinum group, which induces DNA damages and subsequent apoptosis [278]. Importantly, both compounds also exert similar DNA-damaging effects on mtDNA, inducing mitochondrial ROS production and apoptosis [279,280]. Specifically, the treatment of ovarian cancer with cisplatin is known to induce mitochondrial ROS and disrupt the mitochondrial membrane, leading to the release of caspases and apoptosis [279]. Cisplatin can also induce an increase in the expression of the mitochondrial transcription factor, TFAM [279]. In laryngeal squamous cell carcinoma cells, carboplatin is shown to induce apoptosis via the upregulation of cytochrome c and PARP, as well as caspases 3, 8, and 9 [281].

ONC201 and its derivatives bind to CLPP, a protease in the matrix, and induce a proteotoxicity stress response, which eventually triggers mitochondrial failure and apoptosis [204]. As a single agent, ONC201 is capable of suppressing tumors with high expression of CLPP, including certain types of gliomas, endometrial cancer, prostate cancer, mantle cell lymphoma, and adrenal tumors [282]. One of its mechanisms of action is to inactivate the AKT/ERK stress response, which triggers the proapoptotic TNF-related apoptosis-inducing ligand in a wide range of cancer cell lines, including colon cancer and glioblastoma [303].

Clodronate is a pro-drug, which needs to be metabolized by the cell to generate the compound AppCCl_2_p to inhibit the adenine nucleotide transporter (ANT) in mitochondria [285]. ANT inhibition subsequently leads to the disruption of the mitochondrial membrane potential and eventually results in apoptosis. Clodronate is shown to be effective against breast cancer as well as its bone metastases [283,284].

Chlorambucil, another anti-cancer drug, disrupts normal mitochondrial function by alkylating mtDNA and subsequently inducing apoptosis [286,287]. Chlorambucil’s potent ability to induce mtDNA damages allows its clinical application in treating cisplatin-resistant cancers [288]. Although chlorambucil typically targets nDNA, with the addition of mitochondria-penetrating peptides (MPP), such as tri-phenyl phosphonium, it can traverse the mitochondrial membranes and attack mtDNA. In a similar manner, MPPs can be attached to other anti-cancer drugs to enable them to target the mitochondria effectively [304].

### 5.3. Mitochondria-Targeting Molecules

Tri-phenyl phosphonium (TPP) and its derivatives are lipophilic compounds that can be attached to anti-cancer drugs to act as mitochondria-targeting signals, thus facilitating their entry into the mitochondria [305]. Interestingly, TPP is capable of selectively targeting mitochondria of cancer stem cells, providing unique opportunities to investigate these cancerous mitochondria [306]. TPP has been tested for its conjugation to anti-cancer drugs, such as paclitaxel, and delivery to the mitochondria in HeLa cells in vitro and in murine mammary carcinomas in vivo, each showing promising efficiency and increased targeting [307,308].

Szeto-Schiller peptides and their derivatives also serve as attractive candidates for cancer drug delivery [309]. The Szeto-Schiller peptide can transverse the OMM and concentrate in the IMM. Its structure contains alternating sequences of amino acids and basic residues (e.g., tyrosine and dimethyl-tyrosine), enabling its ability to scavenge ROS and reduce their levels [290]. Its derivative, SS-31 (AKA Bendavia), has been shown to act like a drug, which targets cardiolipin, a phospholipid of the IMM that interacts with cytochrome c and can treat heart failure [310,311,312]. Interestingly, SS-31 can protect mitochondrial cristae through its interaction with cardiolipin, suggesting its possible function in maintaining mitochondrial morphology [180]. While the use of Szeto-Schiller peptides reinforces the important role of mitochondria in disease in general, further research could exploit the ability of SS-31′s derivatives in targeting cancers.

Pyridinium-based compounds represent another class of agents that selectively targets the mitochondria of cancer cells. In a hepatoblastoma cell line, pyridinium-substituted tetraphenylethylenes were shown to target the IMM, induce mitochondrial damages, and provoke cancer cell death through increased ROS, decreased membrane potentials, and impaired mitophagy [313]. Importantly, those with longer alkyl chains were proven to induce more morphological changes on the IMM [313]. Another example is the pyridinium and indole-linked F-16 compound, which damages mitochondria and exhibits efficacy against a broad range of cancers, as demonstrated by preclinical and clinical studies [314]. Although pyridinium salts can also target the ER, their mechanisms of action remain unclear [315].

Indolinium-based compounds, such as IR-780, can also target mitochondria. Both in vitro and in vivo studies show that IR-780 induced cancer cell apoptosis by targeting their mitochondria, including drug-resistant lung cancer cells [316]. IR-780 treatment in these cancer cells led to an increase in ROS and the depolarization of the mitochondrial membrane. Another indolinium-based compound is IR-Pyr, which is the intermediate product of an indole-based compound and pyridine, which serves as a part of photodynamic therapy—a light-based cancer treatment. IR-Pyr interacts with hyaluronic acid, a polysaccharide in the mitochondria capable of binding to hyaluronic acid receptors often overexpressed in cancer cells, and this interaction leads to the formation of aggregates detrimental to cancer cells [317].

## 6. Future Perspectives

Mitochondrial proteostasis and integrity are the foundation of cellular health, and dysfunctional mitochondria are intimately linked with human diseases including cancer [14]. Multiple pathways of MPQC exist at the molecular, organellar, and cellular level, seeking to monitor protein quality and repair damaged mitochondria. First, an MPQC surveillance mechanism hovers over mitochondrial precursors during their cytosolic translation and mitochondrial import. The mitoTAD, mitoCPR, and mitoRQC shape the mitochondrial proteome through steady-state turnover of mitochondrial precursors to ensure an appropriate stoichiometry between proteins encoded by nDNA and mtDNA, as well as their proper localization. Second, a large collection of chaperones and proteases reside inside mitochondria, working together to ensure protein folding and quantity. Third, excessive levels of misfolded and unfolded proteins in the mitochondrial matrix or a mito-nuclear protein imbalance activates a conserved UPR^mt^, which functions to selectively induce a transcriptional response aimed at restoring mitochondrial proteostasis. In the event that the mitochondria cannot be repaired, options exist either to eliminate part of damaged mitochondria via MDVs or to remove the entire organelle through mitophagy. Elimination of the entire mitochondria is likely a last resort because it requires the cell to replace these damaged mitochondria.

As a metabolic disease, dysfunctional mitochondria are commonly observed in cancer. Despite almost century-long efforts in investigating the role of mitochondria in tumorigenesis, few anti-cancer drugs can effectively target mitochondria. Recently, altered MPQC has been linked with mitochondrial proteotoxicity and cancer. When hyperactivated, downregulated, or inactivated through mutations, many MPQC players are found to promote tumorigenesis, while others exert tumor suppressive or dual effects. In addition, a number of compounds have been tested for their ability to target specific MPQC regulators, demonstrating promising preclinical and/or clinical efficacy.

Despite our understanding of MPQC in regulating mitochondrial proteins and health, much of this knowledge was gathered through yeast studies. To effectively and safely translate these findings to bedside, further investigation and validation in higher eukaryotes, especially in those that can generate tumors, are needed. The development of novel technology to study and track live mitochondria and the application of innovative in vivo model systems can hasten this progress. A recent study demonstrated the feasibility of this approach, using positron emission topography and radiolabeled 4-fluorobenzyl-triphenylphosphonium to track the mitochondrial membrane in a mouse tumor model [318]. Given the established role of MPQC in cancer, MPQC-targeting compounds could provide opportunities for therapeutic testing, alone or in combination with standard therapy, to treat multiple types of cancer, as well as serving as tools to investigate mitochondrial biology. Undoubtedly, the development of these compounds and continued investigation of the fundamental mechanisms underlying MPQC in mitochondrial health and tumorigenesis hold promise to a new generation of anti-cancer drugs.

## Figures and Tables

**Table 1:** MPQC players involved in tumorigenesis.

Genes	Genetic Alterations	Effects on Tumor	Cancer Context	Mechanisms of Action	Targetability	References
*MRPS16*	Overexpression	Promoting	Glioma	Promotes glioma cell growth, migration, and invasion by the activating the PI3K/AKT/Snail axis	Potential	[138,139]
*MRPS22*	Overexpression	Promoting	Non-small cell lung cancer, breast cancer	Facilitates protein synthesis	ND *	[140,141]
*mtHSP70/Mortalin*	Overexpression	Promoting	Breast cancer, ovarian cancer	Inactivation of p53 and deregulation of apoptosis	Yes	[21]
*MEP*	Overexpression	Promoting	Acute myeloid leukemia (AML)	Interacts with LETM1 (respiratory super complex regulator), a possible inhibitor of cancer	Potential	[142,143]
*HSP70*	Overexpression	Promoting	Breast, endometrial, gastric, leukemia, hepatocellular carcinoma, prostate, colorectal, lung, ovarian, melanoma	Cytosolic protein quality control, apoptosis	Yes	[144,145,146,147,148,149]
*MIA40/CHCHD4*	Overexpression	Promoting	Glioma, osteosarcoma, breast, colon, renal cancer	Oxidoreductase, interacts with p53, and correlates with hypoxic gene regulation that promotes tumorigenesis	ND	[26,150,151]
*HSP90*	Overexpression	Promoting	Hepatocellular carcinoma, squamous cell carcinoma, breast cancer, leukemia, glioblastoma, lung adenocarcinoma	Cytosolic protein quality control, apoptosis	Yes	[24,152,153,154,155]
*POLG1*	Overexpression and mutations	Promoting	Breast, pancreatic cancer, melanoma, colorectal cancer, renal carcinoma	Replicates and repairs mtDNA	Yes	[156,157,158]
*ICT1*	Overexpression	Promoting	Colorectal cancer, prostate, breast, lung, and gastric cancer	Hydrolyzes peptidyl-tRNAs from stalled mitoribosomes	Yes	[68,138,159,160,161,162,163,164]
*TRIT1*	Mutation (single nucleotide polymorphism)	Suppressing	Lymph node metastasis in gastric cancer, lung cancer	tRNA binding to ribosome	No	[165,166,167]
*LARS2*	Downregulation	Suppressing	Primary nasopharyngeal carcinoma	Protein synthesis, catalyzes charged leucine tRNA	ND	[168]
*TOM70*	Altered interaction	Suppressing	Breast cancer	Interaction with RL2 may lead to cell death in breast cancer cells, cytosolic protein import quality	Potential	[169]
*TOM5*	Altered interaction	Suppressing	Non-small cell lung cancer	Interaction with p53 is suggested to inhibit cancer cell proliferation and stability of TOM	Potential	[170]
*PARK2*	Mutations	Suppressing	Glioblastomas, neuroblastomas, lung, breast, and ovarian cancer	Ubiquitination, mitophagy, MDVs	Yes	[171,172,173]
*OMA1*	Downregulated	Suppressing	Breast cancer, possibly others OPA1-relevant cancers	Cleaves proteins such as OPA1, associated with apoptotic pathway	Potential	[174]
*LACTB*	Downregulated	Suppressing	Breast cancer, colorectal, gliomas, hepatocellular carcinomas	Regulates complex I of the ETC	Potential	[165,166,175,176,177]
*ANT1*	Downregulated	Suppressing	Breast cancer, rhabdomyosarcoma	Catalyzes exchange of mitochondrial ATP for cytosolic ADP	Yes	[178,179,180]
*PITRM1*	N/D	Suppressing	Breast cancer, kidney renal clear cell carcinoma, lung adenocarcinoma	Regulates Kruppel-like factor 6 gene, which is a tumor suppressor	ND	[167,181]
*HTRA2 or OMI*	Upregulation Downregulation	Promoting Suppressing	Stomach cancer, thyroid cancer, hepatocellular carcinoma AML	A serine protease in the IMS, interacts with PHB to impact cell proliferation, apoptosis, and mitochondrial proteostasis	Yes	[182,183]
*HSP40*	Upregulation Downregulation	Promoting Suppressing	Breast cancer, CRC, gastric cancer, lung cancer Breast cancer, lung cancer, esophageal squamous cell carcinoma	Helps transport proteins from cytosol to mitochondria	Yes	[27,184,185,186,187,188,189]
*HSP60*	Upregulation Downregulation	Promoting Suppressing	Leukemia, breast, lung, liver, glioblastomas, prostate, ovarian, neuroblastoma, neuroblastomas, cervical, head and neck, colorectal, adrenocortical, pancreatic, hepatocellular carcinoma, pancreatic ductal adenocarcinoma Clear cell renal cell carcinoma	Forms pro-survival complexes in cancers, stabilizes proteins in the matrix	Yes	[89,190,191,192,193,194,195,196,197]
*PHB*	Upregulation Downregulation	Promoting Suppressing	Breast cancer, gall bladder, ovarian cancers Gliomas, nasopharyngeal, and hepatocellular carcinomas, both in gastric cancers	Cell cycle regulation, apoptosis, mitochondrial stability	ND	[198,199,200,201]
*RMND1*	Upregulation Downregulation	Promoting Suppressing	Breast cancer, chronic myeloid leukemia Breast cancer	Assembly of mitoribosome	ND	[178,179,202]
*CLPP*	Upregulation Downregulation	Promoting Suppressing	AML, breast cancer Gastric adenocarcinoma	Degrades damaged or misfolded proteins	Yes	[203,204,205,206,207]
*UQCRC1*	Upregulation Downregulation	Promoting Suppressing	Breast cancer, ovarian cancer, pancreatic ductal adenocarcinoma osteosarcoma CRC, clear cell renal cell carcinomas, gastric cancer	Cleaves precursors that enter mitochondria and key ETC subunits	ND	[208,209,210]
*UQCRC2*	Upregulation Downregulation	Promoting Suppressing	CRC Gliomas	Cleaves precursors that enter mitochondria and key ETC subunits	ND	[211,212]

* ND, Not done.

**Table 2:** Compounds targeting MPQC players in cancer.

Type of Compounds	Drug Name	Target	Mechanisms of Action	Cancer Types	Stage of Drug Development	References
Inhibitors of cytosolic chaperones	DMAG	HSP90	Inhibits HSP90 by competing for ATP binding site and prevents chaperone role	Leukemia, melanoma, breast and ovarian cancers, medulloblastoma, cervical cancer, multiple myeloma, lung cancer	Clinical trials completed	[271,272]
	PES	HSP70	Inhibits HSP70 by acting on its C terminal binding domain, prevents substrate binding, and induces ROS	Lymphoma	Preclinic studies	[273,274]
	Gamitrinibs	HSP90	Binds HSP90 that accumulates at mitochondria and allows for cytochrome c release	Prostate	Completed	[275]
	Nab-paclitaxel	Microtubules	Binds to and stabilizes microtubules, preventing their depolymerization thus inhibiting cellular motility, mitosis, and replication	Non-small lung cell carcinoma	Active, not recruiting, phase 2	[276]
Inhibitors of mitochondrial proteins	Carboplatin	HSP40, DNA	Possible connection with HSP40; used in combinations with other drugs; induces intra-strand and inter-strand DNA cross-links, as well as DNA-protein cross-links. These carboplatin-induced DNA and protein effects result in apoptosis and cell growth inhibition	Non-small lung cell carcinoma, esophageal adenocarcinoma	Active, not recruiting, phase 2	[277,278,279,280,281] NCT02716038; NCT02998268
	ONC201/ONC212 (derivative of ONC201)	CLPP	Hyperactivates CLPP, which leads to protein synthesis inhibition and growth inhibition, or apoptosis via tumor-necrosis-factor-alpha-related apoptosis ligand	AML, gliomas, cervical, breast, endometrial, myeloma, lymphoma, endocrine, solid tumors	Completed (solid tumors), ongoing testing in phase 1 to phase 3	[204,282]
	AppCCl2P (metabolite of clodronate)	ANT	Inhibits ANT (adenine nucleotide transporter), mitochondrial oxygen consumption, and depolarizes mitochondrial membrane, all leading to apoptosis	Breast, bone, prostate, neoplasm	Phase 3, completed	[283,284,285]
	Chlorambucil	mtDNA	Chlorambucil with cisplatin attacks cisplatin-resistant cancer cells, alkylates mtDNA, induces apoptosis through mtDNA damage and mitochondrial metabolic pathways by reducing dependency on glucose, depolarizes mitochondrial membrane, and mtDNA alkylation-induced ROS	Prostate, breast	Completed	[286,287,288]
Targeting mitophagy	LCL-461, liensinine, chloroquine, and hydroxychloroquine, mito-metformin, mito-carboxyl-proxyl-nitroxide		Prevents fusion of autophagosomes with lysosomes and incurs damage to promote apoptosis	Glioma, multiple myeloma, melanoma, lung, pancreatic cancer, sarcoma	Completed	[270,289]
Mitochondria-targeting molecules	Derivatives of TPP	N/A *	Functions as a mitochondrial targeting signal	N/A	N/A	[277]
	Szeto–Schiller peptides—dimethyltyrosine	N/A	Targets molecules to IMM and reduces ROS via its residues	N/A	N/A	[281,283,290]
	Pyridinium-substituted tetraphenylethylene	N/A	Targets IMM, increases ROS, and disrupts membrane potential	N/A	N/A	[286,287,288]
	Indolinium-based compounds	N/A	Increases ROS and depolarizes membrane potential	N/A	N/A	[289,291]

* N/A, not applicable.

## Data Availability

Not applicable.
